# Tai Chi modulates resting alpha and theta EEG rhythms in female individuals with amphetamine-type stimulant dependence during compulsory detoxification

**DOI:** 10.3389/fpsyt.2026.1904447

**Published:** 2026-08-26

**Authors:** Ruzhen Wang, Jingyi Chen, Dong Zhu

**Affiliations:** 1 School of Wushu, Shanghai University of Sport, Shanghai, China; 2 College of Physical Education, Chengdu University of Technology, Chengdu, China; 3 Shandong Province Yantai City Huang-Bohai New Area Guxian No.2 Primary School, Yantai, China

**Keywords:** alpha rhythm, amphetamine-type stimulant, electroencephalography, Tai Chi, theta rhythm

## Abstract

**Background:**

Amphetamine-type stimulants (ATS), primarily methamphetamine, are currently the most frequently abused illicit drugs in China, causing profound neurotoxicity and cognitive impairment, particularly in women. While exercise-based rehabilitation such as Tai Chi has shown benefits for sleep, mood, and executive function, its specific neurophysiological mechanisms in addiction recovery remain unclear.

**Methods:**

Thirty-two female individuals with ATS dependence from a compulsory rehabilitation center were randomly assigned to a Tai Chi group (n=16) or a matched-intensity control group (routine physical rehabilitation; n=16). The intervention lasted 6 months (three 60-min sessions/week). Resting-state electroencephalography (EEG) was recorded pre-intervention, post-intervention, and immediately after a 20-min acute exercise bout. Alpha (7.5–12.5 Hz) and theta (4–7.5 Hz) power were analyzed across 15 scalp electrodes. Of the 32 randomized participants, 25 completed the full 6-month intervention (Tai Chi: n=14; Control: n=11), and 24 participants (Tai Chi: n=13; Control: n=11) provided usable EEG data at all assessment points and were included in the primary neurophysiological analyses.

**Results:**

After the 6-month intervention, both groups exhibited significant reductions in resting alpha and theta power across multiple cortical regions (p<0.05), indicating a general modulation and reduction of cortical oscillatory activity. Acutely, however, Tai Chi elicited significantly higher alpha and theta power in widespread frontal, parietal, temporal, and occipital regions compared to treadmill exercise (p ≤ 0.05). Notably, these acute comparisons reflect post-exercise group differences rather than within-group changes from an acute pre-exercise baseline, as no pre-exercise EEG was recorded immediately before the acute bout.

**Conclusion:**

Long-term moderate-intensity exercise effectively modulates resting EEG rhythms among female individuals with ATS dependence. Furthermore, an acute bout of Tai Chi is associated with a distinct pattern of EEG changes consistent with greater cortical relaxation and internal focus compared to conventional exercise. These findings suggest that Tai Chi serves as a neurophysiologically meaningful, low-cost adjunctive therapy for cognitive and emotional rehabilitation in compulsory detoxification settings. However, the absence of a non-exercise control group, healthy normative data, and immediate pre-exercise EEG recordings precludes causal attribution of these changes solely to Tai Chi or definitive claims of physiological ‘normalization’.

## 1 Introduction

Methamphetamine trafficking and use remain highly prevalent across East Asia, Southeast Asia, and North America, and with inadequate treatment coverage for substance use disorders worldwide (1), this poses a severe threat to public health and safety. In China, amphetamine-type stimulants (ATS), predominantly methamphetamine, have surpassed heroin to become the most frequently abused illicit drugs (2). Chronic ATS use can induce severe cardiovascular dysfunction and persistent neurotoxicity—particularly in the prefrontal cortex, orbitofrontal cortex, and other cortical regions—resulting in significant behavioral and cognitive impairments (3). Women typically progress from initial drug use to full addiction more rapidly than men and are more susceptible to associated psychiatric comorbidities, highlighting the urgent need for accessible, non-pharmacological interventions tailored to female populations.

Tai Chi, a traditional mind-body exercise integrating Chinese medicine and physical movement, is widely practiced globally and has demonstrated efficacy in improving sleep quality (4), mood states (5), postural balance, and executive function in healthy adults and individuals with substance use disorders. Through its emphasis on relaxed yet upright posture, conscious movement control, and seamless coordination of breathing and attention, Tai Chi enhances cardiac function and facilitates emotional regulation. As a moderate-intensity exercise, Tai Chi modulates cortical activity within prefrontal, motor, and occipital networks (6). EEG oscillations, particularly alpha and theta rhythms, are closely associated with arousal states, affective states, and cognitive processing. Enhanced alpha activity during relaxation is typically accompanied by reduced cortical activation and decreased anxiety, whereas theta oscillations are linked to internalized attention, working memory, and meditative states. However, it remains unclear whether the relaxed state and positive affect induced by Tai Chi can functionally counteract drug-related neural alterations in ATS-dependent individuals.

Exercise-based rehabilitation is increasingly recognized as a promising, low-cost adjunct to pharmacological and psychosocial interventions, with consistent evidence demonstrating that exercise reduces craving, improves mood, and enhances executive control in addicted populations (7). Despite these behavioral benefits, the specific neurophysiological mechanisms underlying Tai Chi’s anti-addiction effects remain insufficiently elucidated. Building upon prior research demonstrating that Tai Chi can improve inhibitory control, maintain cognitive flexibility, promote sleep quality, and reduce relapse rates in female ATS-dependent individuals, the present study employed EEG to investigate the acute and long-term effects of Tai Chi on alpha and theta power. Specifically, we recruited a cohort of female ATS-dependent individuals undergoing compulsory isolation treatment; by comparing Tai Chi training with a matched-intensity conventional physical rehabilitation regimen, we aimed to clarify how Tai Chi’s unique movement and breathing patterns facilitate cortical recovery, thereby providing mechanistic evidence for integrating Tai Chi as a safe and feasible rehabilitation strategy within compulsory detention centers.

We hypothesized that: (1) following the 6-month intervention, both exercise groups would exhibit significant reductions in resting alpha and theta power, and Tai Chi practice would be associated with distinct patterns of cortical rhythm modulation compared to conventional rehabilitation. and (2) during the acute phase, a single Tai Chi session would be associated with higher post-exercise alpha and theta power than intensity-matched treadmill exercise, reflecting a state that is simultaneously relaxed and attentively focused.

## 2 Methods

### 2.1 Participants and study design

The protocol was approved by the Ethics Committee of Shanghai University of Sport (Approval No. [SUS]2017024).In 2019, female individuals with ATS dependence were recruited from a women’s compulsory isolation drug detoxification center in Shanghai, China. participants provided written informed consent prior to enrollment. Given the compulsory rehabilitation context, special safeguards were implemented to ensure voluntary participation: (i) recruitment was conducted by independent research staff without disciplinary authority; (ii) participants were explicitly informed that refusal or withdrawal would not affect their rehabilitation duration, institutional privileges, legal status, or access to medical care; and (iii) an independent counselor was present during the consent process to witness and confirm the absence of coercion.

Inclusion criteria were: (1) diagnosis of psychoactive substance-induced dependence (ATS as the primary substance) according to the Chinese Classification and Diagnostic Criteria of Mental Disorders (CCMD-3); (2) female sex, aged 18–60 years; (3) at least primary school education; (4) no history of psychotic disorders, epilepsy, severe cardiovascular disease, or major organic brain lesions; (5) at least 3 months of abstinence from methamphetamine with negative urine toxicology screens; (6) no prior systematic experience with Tai Chi, Qigong, meditation, or similar mind-body practices; and (7) voluntary participation with signed informed consent and capacity to comply with study procedures.

Exclusion criteria were: occurrence of severe discomfort or adverse events warranting withdrawal; poor exercise adherence; premature discharge or logistical barriers preventing completion of the intervention and assessments; and uncontrolled medical conditions arising during the study. Additional exclusion criteria for the neurophysiological analyses included: co-occurring substance dependence (except nicotine), current use of psychotropic medications, pregnancy, or prior Tai Chi training exceeding 3 months within the past 2 years.

**Figure 1:**
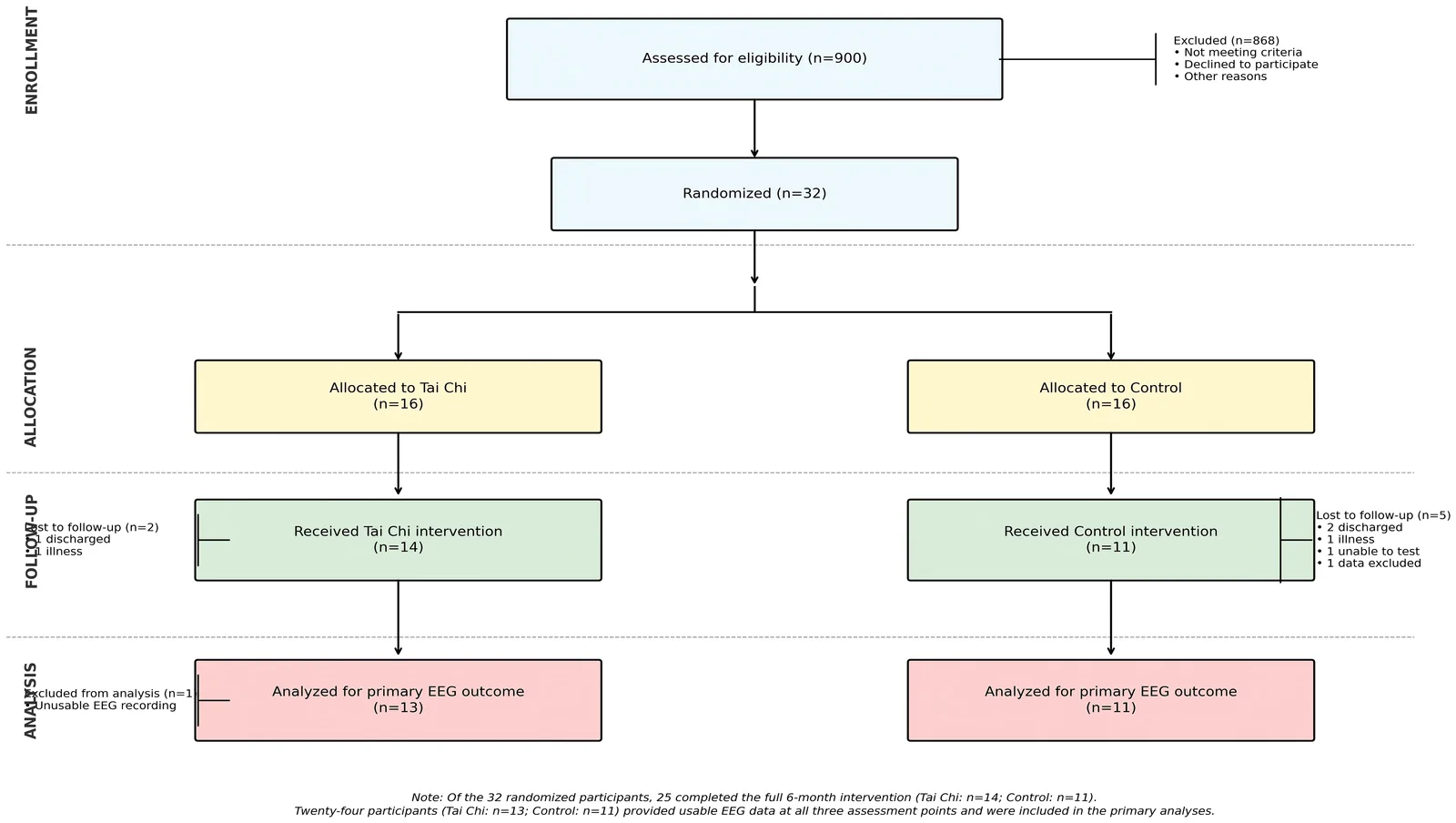
CONSORT flow diagram.

Thirty-two eligible females were randomly assigned to the Tai Chi group (n=16) or the control group (n=16) using a computer-generated randomization sequence with block sizes of 4, stratified by age (< 30 vs. ≥ 30 years) and duration of dependence (< 3 vs. ≥ 3 years). Allocation concealment was maintained using sequentially numbered, opaque, sealed envelopes, opened by an independent research coordinator after baseline assessments. Over the 6-month intervention period, 8 participants withdrew: 4 were discharged from the facility, 2 withdrew due to illness, and 2 had unusable EEG recordings. Consequently, 25 participants completed the full intervention (Tai Chi: n=14; Control: n=11), and 24 participants (Tai Chi: n=13; Control: n=11) provided complete EEG data at all three assessment points and were included in the primary neurophysiological analyses. Demographic characteristics are reported for the 25 participants who completed the intervention, whereas all EEG analyses are based on the 24 participants with complete neurophysiological data (**Figure 1**).

Given the small sample size and the nature of missing data (entirely missing follow-up assessments rather than item-level missingness), we conducted complete-case analyses for primary outcomes.

Baseline assessments included demographic and drug-use questionnaires, physical fitness tests (height, weight, sit-and-reach, one-leg standing with eyes closed, vital capacity, grip strength, blood pressure), and resting-state EEG (Test 1). Following the 6-month intervention, participants completed post-intervention resting-state EEG (Test 2) and an acute exercise plus immediate post-exercise EEG assessment (Test 3: a 24-min Tai Chi or treadmill protocol).

### 2.2 Exercise intervention

The Tai Chi intervention comprised three progressively difficult exercise sequences (Beginner, Intermediate, Advanced) adapted from the standardized 24-form Tai Chi by experts at the Shanghai University of Sport. Each session was conducted three times per week for 60 minutes, consisting of foundational skill practice, form training, and relaxation stretching. Heart rate was monitored continuously using Polar heart rate monitors (Polar Electro Oy, Finland), and exercise intensity was maintained at approximately 50%–60% of age-predicted maximum heart rate (220 − age). Attendance was recorded at each session; the average attendance rate was 85.3% in the Tai Chi group and 82.7% in the control group.

The control group performed routine group physical rehabilitation under supervision, three times per week for 60 minutes per session, including radio calisthenics, finger exercises, treadmill walking/running, and stationary cycling. Heart rate was similarly monitored and maintained at 50%–60% of maximum heart rate to match the Tai Chi group.

For the acute exercise protocol (Test 3), Tai Chi participants performed a 24-min Advanced routine (2-min warm-up using the Intermediate routine, 20-min Advanced routine practice, and 2-min cool-down), whereas control participants completed 24 min of treadmill running at a constant speed (2-min warm-up, 20-min main phase, and 2-min cool-down). Heart rate in both groups was maintained at 50%–60% of maximum heart rate. Immediately following exercise cessation, participants were seated in the EEG recording room, and EEG data acquisition commenced within 3–5 minutes using the same procedure as Tests 1 and 2.

### 2.3 Physical fitness assessment

Physical fitness was measured using the BW-TC-9201L system (Shanghai Bangwen Electronic Technology Co., Ltd.), including height, weight, blood pressure, vital capacity, sit-and-reach flexibility, one-leg standing duration with eyes closed, and grip strength. These tests were administered by trained assessors at baseline and post-intervention.

### 2.4 EEG recording and preprocessing

EEG was recorded using a BrainAmp Standard amplifier and a 64-channel EEG cap (Brain Products GmbH), with electrodes positioned according to the extended 10–20 system. Signals were online referenced to FCz (with AFz as ground), offline re-referenced to the averaged mastoids (TP9/TP10), and FCz was reconstructed as an active channel. Vertical and horizontal electrooculograms were recorded from electrodes placed below the right eye and lateral to the left eye, respectively. Impedances were kept below 10 kΩ using Nuprep abrasive gel and conductive paste. EEG was continuously recorded at a 500 Hz sampling rate in a quiet, dimly lit rehabilitation room, with participants seated and instructed to minimize body movement.

Preprocessing was performed using BrainVision Analyzer 2.0 software. Data were band-pass filtered at 0.01–40 Hz (IIR filter) and notch-filtered at 50 Hz. Bad or unused channels were removed, and ocular artifacts were corrected using independent component analysis (ICA). On average, 2.3 ± 1.1 ICA components were removed per participant based on their topographical distribution and time-course correlation with the EOG channels. Remaining artifacts were rejected following visual inspection. Clean EEG data were segmented into 2-s epochs (50% overlap) using a Hanning window. Only artifact-free epochs were included; a minimum of 60 epochs (approximately 2 minutes) were required per condition for a participant’s data to be retained. Spectral power was computed via Fast Fourier Transform (FFT) with a frequency resolution of 0.5 Hz.

Alpha (7.5–12.5 Hz) and theta (4–7.5 Hz) power (μV²) were extracted from 15 electrodes (F3, F4, F7, F8, C3, C4, P3, P4, P7, P8, T7, T8, Cz, Pz, Oz). These 15 electrodes were selected *a priori* to provide broad cortical coverage across frontal, central, parietal, temporal, and occipital regions while maintaining consistency with prior Tai Chi and addiction EEG literature (6, 8, 9).

During each EEG assessment, 2 minutes of eyes-open and 2 minutes of eyes-closed resting data were collected; spectral power analyses focused on these resting periods. Test 1 was conducted at baseline; Test 2 was conducted after the 6-month intervention; Test 3 was conducted immediately after the 24-min acute exercise protocol. Eyes-open and eyes-closed conditions were analyzed separately; however, because no significant condition × group interactions were observed, the reported values represent the averaged power across both conditions to enhance signal stability, consistent with prior resting-state EEG studies.

### 2.5 Statistical analysis

All analyses were conducted using SPSS 23.0. Independent-samples t-tests and chi-square tests were used to compare baseline demographic and physiological characteristics between groups. Because EEG power data typically exhibit positive skewness, absolute power values were log-transformed (natural logarithm) prior to parametric analyses to improve normality and homogeneity of variance (10).

For longitudinal changes from Test 1 to Test 2, we employed analysis of covariance (ANCOVA) with baseline (Test 1) EEG power as the covariate, intervention group as the between-subjects factor, and post-intervention (Test 2) EEG power as the dependent variable. This approach accounts for baseline imbalances and increases statistical precision. When significant effects were detected, Bonferroni-corrected *post-hoc* comparisons were applied. To control for multiple comparisons across the 15 electrodes, false discovery rate (FDR) correction was applied separately for each frequency band (alpha and theta) and each analysis phase (longitudinal and acute), with a q-value threshold of 0.05.

For acute effects (Test 3), independent-samples t-tests were used to compare alpha and theta power between the Tai Chi and control groups at each electrode. Effect sizes are reported as Cohen’s d for t-tests and partial eta-squared (ηp²) for ANCOVA, with 95% confidence intervals (CIs) where applicable. Exact p-values are reported except when p<0.001.

Assumptions for parametric tests were verified: normality was assessed using Shapiro-Wilk tests, homogeneity of variance using Levene’s tests, and sphericity using Mauchly’s tests. Where sphericity was violated, Greenhouse-Geisser corrections were applied. Statistical significance was set at p<0.05 (two-tailed). Data are presented as mean ± standard deviation (SD).

## 3 Results

### 3.1 Baseline characteristics and physical fitness

#### 3.1.1 Participant characteristics

The Tai Chi and control groups did not differ significantly in age, ethnicity, education level, occupation, marital status, reasons for drug use, drug use frequency, or smoking status (p > 0.05). Overall, the sample exhibited relatively low educational attainment: 64.2% of the Tai Chi group and 72.8% of the control group had junior high school education or less. Non-smokers comprised 28.6% and 9.1% of the Tai Chi and control groups, respectively, while unemployed individuals accounted for 42.9% and 63.6%. Curiosity was the most commonly reported initial reason for drug use, endorsed by 64.3% of the Tai Chi group and 45.5% of the control group. Detailed baseline characteristics are summarized in **Table 1**.

**Table 1:** Demographic characteristics of participants.

Content	Tai Chi group		Control group		x^2^	P Value
	n	%	n	%		
Number of addiction treatment episodes						
1	8	57.1	5	45.5	0.82	0.845
2	4	28.6	3	27.3		
3	1	7.1	1	9.1		
4	1	7.1	2	18.2		
Frequency of drug use						
Occasional use	8	57.1%	3	27.3%	6.506	0.089
Non-daily use	1	7.1%	1	9.1%		
Requires 1–2 daily uses	0	0.0%	4	36.4%		
Three or more times per day	5	35.7%	3	27.3%		
Daily smoking consumption						
No smoking	4	28.6%	1	9.1%	3.973	0.41
4 cigarettes or less	1	7.1%	0	0.0%		
5-10	4	28.6%	3	27.3%		
10-15	2	14.3%	1	9.1%		
Over 16	3	21.4%	6	54.5%		

#### 3.1.2 Baseline physical function

At baseline, the Tai Chi and control groups did not differ significantly on any physical fitness indices (p > 0.05; **Table 2**). Mean age was 28.60 ± 10.34 years in the Tai Chi group and 40.87 ± 8.82 years in the control group (p=0.223, 95% CI [−31.78, 7.24], Cohen’s d=1.29). Mean height was 1.61 m and 1.63 m; mean weight was 61.5 kg and 66.6 kg; BMI was 23.6 and 25.0; vital capacity was 2,077.2 mL and 2,043.3 mL; one-leg standing time was 15.6 s and 17.5 s; grip strength was 27.2 kg and 28.4 kg; sit-and-reach was 10.3 cm and 12.7 cm; resting diastolic blood pressure was 74.7 mmHg and 75.4 mmHg; and systolic blood pressure was 112.5 mmHg and 106.3 mmHg, respectively.

**Table 2:** Independent-samples t-tests for physical fitness before intervention (mean ± SD).

Variable	Tai Chi Group (n=13)	Control Group (n=11)	P value	Cohen’s d	95% CI
Age (years)	28.60 ± 10.34	40.87 ± 8.82	0.223	1.29	[−31.78, 7.24]
Height (m)	1.61 ± 0.07	1.63 ± 0.07	0.532	0.29	[−0.09, 0.05]
Weight (Kg)	61.5 ± 9.55	66.6 ± 8.15	0.863	0.57	[−14.72, 12.52]
Body Mass Index (BMI)	23.6 ± 3.05	25.0 ± 2.79	0.995	0.48	[−5.38, 2.58]
Vital Capacity (ml)	2077.2 ± 605.57	2043.3 ± 584.47	0.637	0.06	[−312.5, 380.3]
Single Leg Stand with Eyes Closed (s)	15.6 ± 10.71	17.5 ± 13.42	0.723	0.16	[−14.36, 10.56]
Hand Grip (Kg)	27.2 ± 5.49	28.4 ± 6.31	0.871	0.20	[−8.44, 6.04]
Sit-and-Reach (cm)	10.3 ± 8.94	12.7 ± 7.60	0.972	0.29	[−9.48, 4.68]
Diastolic Blood Pressure (mmHg)	74.7 ± 13.07	75.4 ± 7.93	0.107	0.06	[−15.26, 13.86]
Systolic Blood Pressure (mmHg)	112.5 ± 16.63	106.3 ± 12.21	0.382	0.42	[−8.48, 20.88]

### 3.2 Baseline EEG alpha/theta differences

We first compared baseline EEG power between the Tai Chi and control groups across 15 scalp electrodes. As shown in **Table 3**, no significant group differences in alpha power were observed at most electrode sites; however, the control group exhibited significantly higher power than the Tai Chi group at P3 (p=0.030) and T7 (p=0.040). For theta power, significant baseline differences were observed at P3 (p=0.020), P4 (p=0.020), P7 (p=0.030), T7 (p<0.001), and Oz (p=0.030), with no significant differences at remaining electrodes (p > 0.05). Overall, these results indicate that the two groups were largely comparable in resting alpha and theta power at baseline, though localized differences existed in parietal, temporal, and occipital regions.

**Table 3:** Results of Independent t-test for Alpha and Theta between the two groups before intervention (Mean ± Sd)(Unit:uV^2^).

Electrode	Alpha			Theta		
	Tai Chi (n=13)	Control (n=11)	P Value	Tai Chi (n=13)	Control (n=11)	P Value
F3	3.17 ± 5.09	2.46 ± 2.90	.42	1.19 ± 1.49	0.81 ± 0.79	.34
F4	3.14 ± 4.34	1.82 ± 2.17	.17	1.17 ± 1.29	0.65 ± 0.69	.20
F7	3.65 ± 4.89	2.72 ± 2.68	.26	1.60 ± 1.67	1.08 ± 0.78	.06
F8	4.20 ± 6.12	2.28 ± 2.39	.06	1.42 ± 1.55	0.82 ± 0.78	.21
C3	6.13 ± 10.98	2.50 ± 2.31	.17	1.88 ± 2.25	1.93 ± 2.70	.41
C4	3.97 ± 3.99	3.18 ± 2.69	.47	2.76 ± 4.40	2.35 ± 2.65	.59
P3	9.90 ± 14.27	2.84 ± 2.90	.03^*^	1.79 ± 1.64	2.76 ± 3.40	.02^*^
P4	8.48 ± 9.87	4.80 ± 2.53	.11	2.14 ± 2.20	1.26 ± 0,62	.02^*^
P7	9.10 ± 14.87	2.80 ± 2.63	.06	1.81 ± 2.37	0.70 ± 0.64	.03^*^
P8	6.05 ± 7.12	3.70 ± 1.46	.12	1.79 ± 1.84	1.68 ± 1.80	.49
T7	4.82 ± 6.11	2.30 ± 1.91	.04^*^	2.80 ± 3.31	0.93 ± 0.71	.00^**^
T8	3.11 ± 5.05	1.60 ± 1.90	.15	1.25 ± 1.44	0.60 ± 0.67	.24
Cz	4.22 ± 6.92	3.24 ± 2.56	.16	2.56 ± 2.32	3.33 ± 3.90	.19
Pz	7.61 ± 13.79	2.80 ± 2.70	.05	1.40 ± 1.70	0.72 ± 0.53	.05
Oz	5.19 ± 6.82	3.06 ± 1.75	.05	1.26 ± 1.33	0.91 ± 0.53	.03^*^

*p<0.05, **p<0.01.

### 3.3 Post-intervention group differences

#### 3.3.1 Post-intervention alpha and theta power differences between groups

Following the 6-month exercise intervention, independent-samples t-tests were conducted on resting-state EEG data from Test 2 to compare alpha and theta power between the Tai Chi and control groups (**Table 4**). For alpha rhythm, significant group differences were observed at P4 (p=0.010), P8 (p=0.010), and Oz (p=0.010), with no significant differences at remaining electrodes (p > 0.05). For theta rhythm, significant group differences were observed at F3 (p=0.010), F4 (p=0.020), C4 (p=0.010), and T7 (p=0.030), with no significant differences at remaining electrodes (p > 0.05). These results suggest that long-term Tai Chi practice, compared to conventional exercise, differentially modulates alpha power in parietal and occipital regions and theta power in frontal, central, and temporal regions.

**Table 4:** Independent t-test of Alpha and Theta rhythms under resting state after intervention (Mean ± Sd)(Unit:uV^2)^.

Electrode	Alpha			Theta		
	Tai Chi (n=13)	Control (n=11)	P Value	Tai Chi (n=13)	Control (n=11)	P Value
F3	0.76 ± 0.67	0.45 ± 0.36	.19	0.24 ± 0.12	0.42 ± 0.38	.01^**^
F4	0.92 ± 0.79	0.77 ± 0.61	.43	0.40 ± 0.26	0.63 ± 0.41	.02^*^
F7	0.92 ± 0.99	0.58 ± 0.41	.04	0.30 ± 0.24	0.44 ± 0.30	.60
F8	1.17 ± 1.29	1.59 ± 1.57	.76	0.61 ± 0.52	1.11 ± 0.78	.13
C3	0.59 ± 0.39	0.49 ± 0.39	.76	0.20 ± 0.12	0.29 ± 0.26	.40
C4	0.61 ± 0.48	0.73 ± 0.68	.23	0.25 ± 0.14	0.47 ± 0.33	.01^**^
P3	1.35 ± 1.03	0.99 ± 0.87	.41	0.27 ± 0.17	0.34 ± 0.32	.59
P4	2.23 ± 2.00	1.07 ± 0.87	.01^**^	0.33 ± 0.20	0.43 ± 0.33	.61
P7	1.26 ± 1.39	0.77 ± 0.70	.38	0.23 ± 0.14	0.32 ± 0.38	.32
P8	2.05 ± 1.92	0.80 ± 0.72	.01^**^	0.30 ± 0.16	0.37 ± 0.29	.28
T7	0.50 ± 0.47	0.88 ± 1.91	.14	0.17 ± 0.11	0.84 ± 1.94	.03^*^
T8	0.66 ± 0.58	0.71 ± 0.52	.67	0.34 ± 0.22	0.56 ± 0.39	.10
Cz	0.48 ± 0.31	0.51 ± 0.46	.23	0.21 ± 0.10	0.34 ± 0.28	.10
Pz	1.76 ± 1.95	0.96 ± 0.73	.18	0.32 ± 0.20	0.38 ± 0.31	.69
Oz	1.74 ± 1.27	0.69 ± 0.62	.01^**^	0.39 ± 0.25	0.38 ± 0.36	.79

*p<0.05, **p<0.01.

To account for baseline EEG imbalances, we conducted ANCOVAs with baseline (Test 1) power as the covariate and group as the between-subjects factor. After adjusting for baseline values, the group differences at P4 (F(1,21)=5.42, p=0.030, ηp²=0.205), P8 (F(1,21)=6.18, p=0.021, ηp²=0.227), and Oz (F(1,21)=7.89, p=0.011, ηp²=0.273) for alpha power remained significant. For theta power, significant adjusted group differences were observed at F3 (F(1,21)=5.12, p=0.035, ηp²=0.196), F4 (F(1,21)=4.89, p=0.038, ηp²=0.189), and C4 (F(1,21)=6.55, p=0.018, ηp²=0.238).

#### 3.3.2 Longitudinal changes in alpha power within groups

To examine longitudinal changes in alpha power, we conducted repeated-measures ANCOVA with time (pre- vs. post-intervention) as the within-subjects factor and group (Tai Chi vs. Control) as the between-subjects factor, using baseline power as the covariate. Significant main effects of time were observed at multiple electrode sites, including F4 (F(1,21)=5.76, p=0.026, ηp²=0.215), F7 (F(1,21)=9.75, p=0.005, ηp²=0.317), C3 (F(1,21)=5.15, p=0.034, ηp²=0.197), C4 (F(1,21)=17.31, p<0.001, ηp²=0.452), P3 (F(1,21)=6.25, p=0.021, ηp²=0.229), P4 (F(1,21)=12.40, p=0.002, ηp²=0.371), P7 (F(1,21)=5.54, p=0.028, ηp²=0.209), P8 (F(1,21)=11.18, p=0.003, ηp²=0.347), T7 (F(1,21)=8.85, p=0.007, ηp²=0.296), T8 (F(1,21)=4.74, p=0.041, ηp²=0.184), Cz (F(1,21)=8.81, p=0.007, ηp²=0.295), and Oz (F(1,21)=9.51, p=0.006, ηp²=0.312). No significant time × group interactions or main effects of group were observed (p > 0.05). These findings indicate that the 6-month exercise intervention—regardless of modality—was associated with significant reductions in resting alpha power across 12 electrode sites in female individuals with ATS dependence. Detailed pre- and post-intervention values are presented in **Table 5**.

**Table 5:** Results of mean power changes of alpha rhythm in subjects after intervention (Mean ± Sd) (Value:uV^2^).

Alpha							
Electrode	Tai Chi group		Control group		Time F value	Group F value	Time×group F value
	Pre-intervention	Post-intervention	Pre-intervention	Post-intervention			
F3	3.17 ± 5.09	0.76 ± 0.67	2.46 ± 2.90	0.45 ± 0.36	7.07	0.32	0.06
F4	3.14 ± 4.34	0.92 ± 0.79	1.82 ± 2.17	0.77 ± 0.61	5.76^*^	0.87	0.75
F7	3.65 ± 4.89	0.92 ± 0.99	2.72 ± 2.68	0.58 ± 0.41	9.75^**^	0.49	0.15
F8	4.20 ± 6.12	1.17 ± 1.29	2.28 ± 2.39	1.59 ± 1.57	3.85	0.47	1.53
C3	6.13 ± 10.98	0.59 ± 0.39	2.50 ± 2.31	0.49 ± 0.39	5.15^*^	1.17	1.13
C4	3.97 ± 3.99	0.61 ± 0.48	3.18 ± 2.69	0.73 ± 0.68	17.31^**^	0.21	0.43
P3	9.90 ± 14.27	1.35 ± 1.03	2.84 ± 2.90	0.99 ± 0.87	6.25^*^	2.56	2.6
P4	8.48 ± 9.87	2.23 ± 2.00	4.80 ± 2.53	1.07 ± 0.87	12.4^**^	2.01	0.79
P7	9.10 ± 14.87	1.26 ± 1.39	2.80 ± 2.63	0.77 ± 0.70	5.54^*^	1.9	1.92
P8	6.05 ± 7.12	2.05 ± 1.92	3.70 ± 1.46	0.80 ± 0.72	11.18^**^	2.12	0.29
T7	4.82 ± 6.11	0.50 ± 0.47	2.30 ± 1.91	0.88 ± 1.91	8.85^**^	1.07	2.26
T8	3.11 ± 5.05	0.66 ± 0.58	1.60 ± 1.90	0.71 ± 0.52	4.77^**^	0.7	1.04
Cz	4.22 ± 6.92	0.48 ± 0.31	3.24 ± 2.56	0.51 ± 0.46	8.81^**^	0.18	0.21
Pz	7.61 ± 13.79	1.76 ± 1.95	2.80 ± 2.70	0.96 ± 0.73	3.74	1.51	1.02
Oz	5.19 ± 6.82	1.74 ± 1.27	3.06 ± 1.75	0.69 ± 0.62	9.51^**^	0.75	0.33

*p<0.05, **p<0.01.

#### 3.3.3 Longitudinal changes in theta power within groups

For theta power, repeated-measures ANCOVA revealed significant main effects of time at F3 (F(1,21)=6.82, p=0.017, ηp²=0.245), F7 (F(1,21)=11.98, p=0.002, ηp²=0.363), C3 (F(1,21)=10.67, p=0.004, ηp²=0.337), C4 (F(1,21)=8.24, p=0.009, ηp²=0.282), P3 (F(1,21)=13.61, p=0.001, ηp²=0.393), P4 (F(1,21)=14.51, p=0.001, ηp²=0.409), P7 (F(1,21)=7.01, p=0.015, ηp²=0.250), P8 (F(1,21)=13.94, p=0.001, ηp²=0.399), T7 (F(1,21)=5.65, p=0.027, ηp²=0.212), T8 (F(1,21)=4.09, p=0.056, ηp²=0.163), Cz (F(1,21)=16.98, p<0.001, ηp²=0.447), Pz (F(1,21)=7.19, p=0.014, ηp²=0.255), and Oz (F(1,21)=10.55, p=0.004, ηp²=0.334). No significant time × group interactions were observed except at T7 (F(1,21)=4.90, p=0.038, ηp²=0.189). These results demonstrate that long-term exercise significantly reduced resting theta power across frontal, central, parietal, temporal, and occipital regions in both groups. Detailed pre- and post-intervention values are presented in **Table 6**.

**Table 6:** Changes in the mean power spectrum of theta rhythm in subjects after intervention (Mean ± Sd) (Unit:uV^2^).

Theta							
Electrode	Tai Chi group		Control group		Time F value	Group F value	Time×group F value
	Pre-intervention	Post-intervention	Pre-intervention	Post-intervention			
F3	1.19 ± 1.49	0.24 ± 0.12	0.81 ± 0.79	0.42 ± 0.38	6.82^*^	0.14	1.23
F4	1.17 ± 1.29	0.40 ± 0.26	0.65 ± 0.69	0.63 ± 0.41	3.25	0.37	2.95
F7	1.60 ± 1.67	0.30 ± 0.24	1.08 ± 0.78	0.44 ± 0.30	11.98^**^	0.44	1.38
F8	1.42 ± 1.55	0.61 ± 0.52	0.82 ± 0.78	1.11 ± 0.78	0.90	0.03	4.12
C3	1.88 ± 2.25	0.20 ± 0.12	1.93 ± 2.70	0.29 ± 0.26	10.67^**^	0.02	0.00
C4	2.76 ± 4.40	0.25 ± 0.14	2.35 ± 2.65	0.47 ± 0.33	8.24^**^	0.02	0.17
P3	1.79 ± 1.64	0.27 ± 0.17	2.76 ± 3.40	0.34 ± 0.32	13.61^**^	0.95	0.71
P4	2.14 ± 2.20	0.33 ± 0.20	1.26 ± 0,62	0.43 ± 0.33	14.51^**^	1.25	2.01
P7	1.81 ± 2.37	0.23 ± 0.14	0.70 ± 0.64	0.32 ± 0.38	7.01^*^	1.84	2.67
P8	1.79 ± 1.84	0.30 ± 0.16	1.68 ± 1.80	0.37 ± 0.29	13.94^**^	0.00	0.06
T7	2.80 ± 3.31	0.17 ± 0.11	0.93 ± 0.71	0.84 ± 1.94	5.65^*^	1.09	4.9^*^
T8	1.25 ± 1.44	0.34 ± 0.22	0.60 ± 0.67	0.56 ± 0.39	4.09	0.73	3.38
Cz	2.56 ± 2.32	0.21 ± 0.10	3.33 ± 3.90	0.34 ± 0.28	16.98^**^	0.49	0.24
Pz	1.40 ± 1.70	0.32 ± 0.20	0.72 ± 0.53	0.38 ± 0.31	7.19^**^	1.21	2.03
Oz	1.26 ± 1.33	0.39 ± 0.25	0.91 ± 0.53	0.38 ± 0.36	10.55^**^	0.57	0.66

*p<0.05, **p<0.01.

### 3.4 Acute Tai Chi vs. treadmill exercise EEG differences

Independent-samples t-tests were conducted on post-exercise EEG data (Test 3) to compare the Tai Chi and control groups immediately following the 20-min acute exercise bout (**Table 7**).

**Table 7:** Independent t-test of Alpha and Theta rhythms between the two groups after acute exercise (Mean ± Sd) (Unit:uV^2^).

Electrode	Alpha			Theta		
	Tai Chi (n=13)	Control (n=11)	P Value	Tai Chi (n=13)	Control (n=11)	P Value
F3	0.98 ± 0.62	0.58 ± 0.42	.09	0.55 ± 0.38	0.44 ± 0.37	.89
F4	1.10 ± 0.85	0.72 ± 0.54	.06	0.69 ± 0.55	0.62 ± 0.52	.81
F7	0.93 ± 0.71	0.58 ± 0.26	.00^**^	0.56 ± 0.40	0.40 ± 0.23	.05^*^
F8	1.70 ± 2.36	0.96 ± 0.55	.05^*^	1.15 ± 1.42	0.74 ± 0.44	.13
C3	0.73 ± 0.50	0.76 ± 0.53	.93	0.43 ± 0.41	0.36 ± 0.28	.93
C4	1.00 ± 0.95	0.65 ± 0.37	.01^**^	0.56 ± 0.62	0.43 ± 0.29	.16
P3	2.46 ± 2.76	1.91 ± 1.68	.29	0.63 ± 0.50	0.47 ± 0.26	.09
P4	3.77 ± 4.01	1.58 ± 1.16	.01^**^	0.91 ± 0.70	0.48 ± 0.26	.01^*^
P7	2.14 ± 2.92	1.42 ± 0.93	.12	0.55 ± 0.52	0.46 ± 0.27	.21
P8	3.23 ± 2.87	1.28 ± 0.77	.00^**^	0.87 ± 0.80	0.47 ± 0.25	.02^*^
T7	0.53 ± 0.38	2.29 ± 6.07	.04^*^	0.31 ± 0.28	4.91 ± 15.34	.03^*^
T8	1.08 ± 0.84	0.56 ± 0.26	.02^*^	0.66 ± 0.52	0.42 ± 0.27	.02^*^
Cz	0.91 ± 0.72	0.53 ± 0.30	.02^*^	0.49 ± 0.48	0.38 ± 0.27	.54
Pz	2.79 ± 2.82	1.65 ± 1.51	.04^*^	0.76 ± 0.60	0.48 ± 0.28	.05^*^
Oz	2.94 ± 2.77	1.06 ± 0.59	.03^*^	0.81 ± 0.53	0.46 ± 0.25	.07

*p<0.05, **p<0.01.

For alpha rhythm, significant group differences were observed at F7 (p<0.001), F8 (p=0.050), C4 (p=0.010), P4 (p=0.010), P8 (p=0.010), T7 (p=0.040), T8 (p=0.020), Cz (p=0.040), Pz (p=0.020), and Oz (p=0.030); the Tai Chi group exhibited higher mean alpha power than the treadmill group at all these electrodes. These results indicate that, compared to continuous moderate-intensity treadmill exercise, a single Tai Chi session elicited more pronounced alpha rhythm enhancement across frontal, central, parietal, temporal, and occipital regions, consistent with a state of greater cortical relaxation and reduced arousal.

For theta rhythm, significant group differences were observed at F7 (p=0.050), P4 (p=0.010), P8 (p=0.020), T7 (p=0.030), T8 (p=0.020), and Pz (p=0.050). The Tai Chi group exhibited higher theta power than the control group at F7, P4, P8, T8, and Pz. However, at T7, the control group exhibited substantially higher theta power (4.91 ± 15.34 μV²) than the Tai Chi group (0.31 ± 0.28 μV²), accompanied by a large standard deviation suggestive of extreme outliers in the control group at this electrode. Excluding the apparent outlier in the control group (one participant with theta power > 40 μV²), the T7 difference was no longer significant (p=0.18). Overall, the pattern suggests that acute Tai Chi training produced higher theta power than treadmill exercise at most posterior and temporal sites, though baseline pre-existing differences between groups may partially contribute to these post-exercise differences.

## 4 Discussion

This study investigated the effects of a 6-month Tai Chi intervention on resting-state alpha and theta EEG power in female individuals with ATS dependence undergoing compulsory isolation treatment, compared to a matched-intensity conventional rehabilitation program. Of the 32 enrolled participants, 24 completed the full EEG assessment protocol. Repeated-measures analyses revealed that both groups exhibited significant reductions in resting alpha and theta power across multiple scalp regions after 6 months, with no significant group × time interactions. In contrast, acute post-exercise assessment demonstrated that Tai Chi elicited significantly higher alpha and theta power than treadmill exercise at multiple electrodes.

### 4.1 Effects of 6-months of Tai Chi practice on alpha rhythm

Alpha activity typically emerges during wakeful, eyes-closed, relaxed states and is closely associated with cognitive function and emotional regulation. The present study demonstrated significant reductions in resting alpha power from pre- to post-intervention in both the Tai Chi and control groups, suggesting that regular moderate-intensity exercise may help reduce elevated alpha activity associated with ATS dependence in women. Previous pharmacological studies in healthy volunteers have shown that amphetamine administration increases EEG power and autonomic arousal, shortens reaction time, and impairs visual memory performance (11), whereas elevated alpha power has also been linked to the euphoric effects of cocaine and cannabis (12, 13). Accordingly, the observed reduction in alpha power after 6 months may reflect a partial attenuation of drug-related cortical hyperarousal; however, without a healthy control group or normative reference values, we cannot definitively conclude that these changes represent a “normalization” of pathological activity.

It is noteworthy that 79.2% of participants in this study were smokers; due to strict management policies at the compulsory rehabilitation center, participants were required to abstain from smoking throughout the intervention period. Previous studies have shown that daily smokers often exhibit lower resting gamma and alpha power and higher impulsivity compared to non-smokers, and that heavy smokers display reduced alpha and increased high-frequency (beta and gamma) activity (14), though some early studies reported acute increases in alpha frequency immediately after smoking (15). In the present sample, despite enforced smoking cessation, resting alpha power decreased significantly over the 6-month period in both groups, making it difficult to disentangle the relative contributions of smoking cessation versus exercise training to alpha modulation. Nevertheless, the consistent downward trend across electrodes suggests that long-term structured exercise—whether Tai Chi or conventional activity—exerts a beneficial regulatory effect on cortical alpha activity in this population, independent of nicotine withdrawal effects.

### 4.2 Effects of 6-months of Tai Chi practice on theta rhythm

Both groups exhibited significant reductions in resting theta power across frontal, central, parietal, temporal, and midline regions after the 6-month intervention, with a significant time × group interaction observed only at T7. From a neurophysiological perspective, addiction is conceptualized as a chronic brain disease characterized by dysfunctional reward and motivation circuitry, leading to intense craving, compulsive use, and high relapse risk (16). In nicotine dependence, theta and beta spectral power have been shown to increase in response to smoking-related cues, and theta activity is elevated during withdrawal but attenuated after cigarette consumption, suggesting that higher theta power may be associated with craving and negative affect during abstinence (17).

In the present study, participants were prohibited from smoking during compulsory rehabilitation; however, as the abstinence period lengthened and participants engaged in exercise training, resting theta power decreased significantly. This finding is consistent with prior research demonstrating that regular aerobic exercise alleviates withdrawal symptoms and reduces drug craving (7). Pharmacological EEG studies have shown that acute cocaine administration rapidly increases theta, alpha, and beta absolute power in prefrontal regions, with theta power negatively correlated with cortical blood flow, suggesting that theta elevation may reflect reduced cerebral perfusion and increased internal tension (18). Conversely, the substantial reduction in theta power observed after long-term exercise in our ATS-dependent participants may suggest improved cortical blood flow and oxygen delivery, potentially mediated by enhanced cardiovascular fitness and circulatory improvements resulting from the aerobic components of the training regimen. However, this interpretation remains speculative because cerebral blood flow was not directly measured.

Comparative studies across the lifespan and in various neurological conditions indicate that higher alpha power—particularly in the upper alpha band—is positively correlated with cognitive performance, whereas elevated theta power is negatively correlated with cognitive ability (19). Patients receiving methadone maintenance treatment or actively using opioids typically exhibit increased theta and beta power compared to healthy controls. A recent meta-analysis reported that Tai Chi confers significant benefits on executive function in older adults (20), possibly due to its combination of aerobic, resistance, and flexibility training components. Given that chronic ATS use impairs prefrontal and striatal neural circuits underlying inhibitory control (21), our finding of concurrent alpha and theta power reductions after long-term exercise supports the hypothesis that exercise—particularly Tai Chi—may contribute to the restoration of cognitive control. However, because no significant time × group interactions were observed in the longitudinal analyses, we cannot conclude that Tai Chi is superior to conventional exercise for producing these long-term EEG changes. Instead, both interventions appear to be associated with comparable longitudinal reductions in resting alpha and theta power.

Animal studies provide convergent evidence for exercise-induced neuroplasticity. In rodents, treadmill running rapidly increases mean EEG frequency and theta power during exercise, with these changes reversing upon cessation, indicating co-activation of the cortex and autonomic nervous system (22). Chronic wheel running has been shown to increase capillary density in the cerebellum, enhance cortical high-affinity choline uptake and dopamine receptor density, upregulate brain-derived neurotrophic factor (BDNF) expression, and promote adult hippocampal neurogenesis (23). These cellular, molecular, and neurochemical adaptations are thought to underlie exercise-related improvements in perception, cognition, and motor function. Consistently, a 9-week Tai Chi intervention (4 sessions/week, 1 hour/session) was reported to increase P300 amplitude and decrease ERN amplitude, indicating enhanced executive control (24), while other studies have shown that long-term Tai Chi training optimizes cortical alpha modulation and improves postural balance in older adults (25).

#### 4.2.1 Integrating chronic and acute findings

A potential paradox emerges from our results: chronic exercise reduced resting alpha/theta power (interpreted as reduced hyperarousal), whereas acute Tai Chi increased alpha/theta power relative to treadmill exercise (interpreted as enhanced relaxation and internal focus). We propose that these findings reflect distinct neurophysiological time scales and mechanisms. The chronic reduction likely reflects long-term neuroadaptive changes—such as improved autonomic regulation, enhanced cerebrovascular efficiency, and reduced withdrawal-related hyperarousal—resulting from sustained abstinence and repeated exercise exposure. In contrast, the acute increase during Tai Chi likely reflects the immediate engagement of attentional and meditative networks: the coordinated breathing, postural control, and internal attention characteristic of Tai Chi may transiently enhance alpha/theta generation in a manner analogous to mindfulness meditation, promoting a state of relaxed alertness that is distinct from the more externally oriented arousal produced by treadmill exercise. Thus, chronic exercise may contribute to lowering baseline cortical excitability over time, while acute Tai Chi transiently elevates specific rhythms above this new baseline to facilitate cognitive and emotional processing. This conceptual framework should be regarded as a mechanistic hypothesis that warrants further empirical testing with direct measures of autonomic regulation and cerebral blood flow.

### 4.3 Acute Tai Chi vs. treadmill exercise: implications for cognitive function

Independent-samples t-tests on post-exercise EEG data (Test 3) revealed that the Tai Chi group exhibited significantly higher alpha power than the control group at F7, F8, C4, P4, P8, T7, T8, Cz, Pz, and Oz, and higher theta power at F7, P4, P8, T7, T8, and Pz. It is critical to note that these analyses compare post-exercise states between groups and do not demonstrate within-group increases from a pre-exercise acute baseline, as no immediate pre-exercise EEG was recorded during Test 3. Therefore, we interpret these findings as reflecting divergent acute neurophysiological states induced by Tai Chi versus treadmill exercise, rather than definitive evidence that Tai Chi “increases” EEG power from an acute pre-exercise baseline.

Our findings are consistent with prior research demonstrating that different Qigong practices (e.g., Wu Qin Xi vs. Liu Zi Jue) produce distinct EEG activation patterns attributable to differences in respiratory regulation and attentional focus (26). In those studies, stronger theta activity during Liu Zi Jue was attributed to sound-guided breathing and internalized attention, whereas Wu Qin Jue imposed fewer respiratory constraints, resulting in a different balance of alpha and theta modulation. Similarly, Tai Chi requires coordinated regulation of breathing with whole-body movement and sustained attention to limb control and postural alignment, which may preferentially enhance internally directed attention and meditative engagement. This mechanism may explain why Tai Chi produced more widespread alpha and theta enhancement than treadmill exercise at equivalent heart rates.

Most studies on acute exercise and EEG have emphasized alpha activity enhancement (27), typically interpreted as reflecting reduced cortical activation, relaxation, fatigue, or decreased anxiety. Following moderate-intensity cycling, increased occipital and parietal alpha activity has been reported, and frontal alpha asymmetry shifts toward relatively greater left prefrontal activity after aerobic exercise have been associated with improved emotional states (28). However, because Tai Chi and treadmill exercise were matched for cardiovascular intensity in the present study, the more pronounced and widespread alpha enhancement observed after Tai Chi suggests that movement complexity, mindful breathing, and attentional focus are critical factors modulating acute EEG responses beyond simple aerobic load.

Evidence from psychology, neurophysiology, and neuroimaging indicates that acute moderate-intensity aerobic exercise transiently enhances multiple dimensions of cognitive function, including attention and executive function, with inhibitory control showing particularly robust benefits that may persist after exercise cessation (29). Meta-analytic evidence confirms that exercise modality, intensity, and duration differentially modulate these acute neurocognitive effects (30). fNIRS studies in Tai Chi practitioners further suggest that short-term Tai Chi training preferentially enhances activation in motor and occipital regions, with relatively limited immediate effects on the prefrontal cortex (31). Combined with our EEG findings, this pattern supports the view that acute Tai Chi training may simultaneously activate sensorimotor and visual networks while promoting a relaxed, internally oriented mental state, creating favorable conditions for improved inhibitory control and emotional regulation in female individuals with ATS dependence.

## 5 Conclusion

Following the 6-month exercise intervention, both groups exhibited significant reductions in resting alpha power at F4, F7, C3, C4, P3, P4, P7, P8, T7, T8, Cz, and Oz, with no significant group × time interactions. Both groups also showed significant reductions in theta power at F3, F7, C3, C4, P3, P4, P7, P8, T7, Cz, Pz, and Oz. These findings indicate that long-term moderate-intensity exercise—regardless of specific modality—can effectively modulate ATS-related cortical activity and can effectively modulate ATS-related cortical activity, and may contribute to neurofunctional recovery in women undergoing compulsory detoxification. The significant reduction in resting theta power further suggests that improved cardiovascular fitness may enhance cerebral hemodynamics and oxygen delivery.

Acutely, a 20-min Tai Chi session produced distinct EEG characteristics compared to treadmill running: Tai Chi significantly increased alpha power at F7, C4, P4, P8, T7, T8, Cz, Pz, and Oz, reflecting reduced cortical hyperactivation and decreased anxiety; it also elicited relatively higher theta power at F7, P4, P8, T8, and Pz, suggesting co-activation of attentional cortical networks and the autonomic nervous system. This unique dual modulation—simultaneously enhancing relaxation and internal attention—renders Tai Chi a promising adjunctive intervention for supporting cognitive rehabilitation in addiction recovery. However, these conclusions are based on observed EEG changes rather than direct measures of cognition, craving, or cerebral blood flow, and should be considered preliminary pending replication in larger, controlled trials.

## 6 Limitations and future directions

Despite recent advances in exercise-based addiction rehabilitation, several limitations must be acknowledged. First, the sample size was small (n=32 randomized, n=24 completers with usable EEG data), constrained by equipment availability, testing space, and the rehabilitation environment. The resulting limited statistical power may have precluded detection of modest between-group differences in longitudinal analyses. Future studies should recruit larger samples and implement longitudinal follow-up after discharge to assess relapse rates and determine whether exercise interventions—particularly Tai Chi—can sustainably reduce relapse risk in ATS-dependent individuals.

Second, significant sex differences in exercise intervention responses have been widely reported, yet this study focused exclusively on female methamphetamine users. Future research should include male methamphetamine-dependent individuals and extend to other substance-dependent populations (e.g., cocaine, cannabis, alcohol) to determine whether Tai Chi produces comparable benefits across drug classes.

Third, we did not collect craving ratings, drug use frequency, or detailed executive function assessments concurrent with EEG monitoring, limiting our ability to directly link neurophysiological changes to addiction-related behavioral and cognitive recovery.

Fourth, the absence of a non-exercise (waitlist or treatment-as-usual) control group precludes definitive attribution of observed changes to exercise or Tai Chi per se, as opposed to spontaneous recovery during prolonged abstinence, institutional structure, nicotine withdrawal, or psychosocial treatment. Both groups experienced compulsory rehabilitation, smoking cessation, and standardized routines; therefore, the observed EEG changes may reflect a combination of exercise, detoxification, and institutional factors. Causal inferences regarding exercise-specific neurophysiological recovery should be interpreted with caution.

Fifth, the control intervention was heterogeneous across the long-term (radio calisthenics, finger exercises, treadmill, stationary cycling) and acute (treadmill only) phases, potentially introducing confounding. Future studies should employ identical control activities for both chronic and acute comparisons.

Sixth, baseline EEG imbalances were observed at several electrodes. Although we employed ANCOVA to adjust for baseline differences, residual confounding cannot be entirely excluded. Future studies should ensure larger sample sizes to minimize random baseline imbalances.

Seventh, the acute comparison (Test 3) lacked an immediate pre-exercise baseline, preventing definitive conclusions about within-group acute changes. Future research should incorporate pre- and post-acute exercise EEG assessments using a crossover or repeated-measures design.

Finally, while we observed significant EEG changes, these neural indices do not independently confirm improvements in cerebral perfusion, neuroplasticity, craving reduction, or cognitive recovery. Such interpretations should be regarded as mechanistic hypotheses requiring direct empirical validation.

## Data Availability

The original contributions presented in the study are included in the article/Supplementary Material. Further inquiries can be directed to the corresponding author.
